# Assessment of childhood immunization services at private healthcare facilities in Indonesia: a case study in a highly-populated city

**DOI:** 10.3389/fpubh.2023.1093387

**Published:** 2023-07-27

**Authors:** Auliya A. Suwantika, Neily Zakiyah, Rizky Abdulah, Ajeng Diantini

**Affiliations:** ^1^Department of Pharmacology and Clinical Pharmacy, Faculty of Pharmacy, Universitas Padjadjaran, Bandung, Indonesia; ^2^Center of Excellence for Pharmaceutical Care Innovation (PHARCI), Universitas Padjadjaran, Bandung, Indonesia; ^3^Center for Health Technology Assessment, Universitas Padjadjaran, Bandung, Indonesia

**Keywords:** vaccination, vaccine, situation analysis, qualitative, interview

## Abstract

**Introduction:**

The need to enhance the utilization of the private sector for immunization programs in Indonesia while maintaining the high quality of services provided is evident. This study aimed to rapidly assess immunization services at private healthcare facilities in Indonesia by using Bandung, the most densely populated city, as the reference case.

**Methods:**

Initially, a situation analysis was conducted by collecting data from selected healthcare facilities (*n* = 9). Furthermore, a qualitative study was taken into account by developing framework approaches and conducting interviews with different layers, such as mid-level managers at healthcare facilities (*n* = 9), professional organizations (*n* = 4), and public stakeholders (*n* = 7).

**Results:**

The situation analysis showed that private healthcare facilities had provided sufficient time for essential childhood immunization services with adequate staff. Nevertheless, the number of limited staff the Ministry of Health (MoH) has trained remains a programmatic problem. Furthermore, private healthcare facilities have used the MoH guidelines and additional internal guidelines for immunization services as the primary reference, including in the efforts to provide complete and reliable equipment. Vaccine availability at private healthcare facilities is manageable with an acceptable out-of-stock level. The results of our interviews highlighted three key findings: the lack of coordination across public and private sectors, the need for immunization service delivery improvement at private healthcare facilities, and the urgency to strengthen institutional capacity for advocacy and immunization systems support.

**Conclusion:**

Even though private healthcare facilities have been shown to make a modest contribution to childhood immunization services in Indonesia, efforts should be made to expand the role of private healthcare facilities in improving the performance of routine immunization programs.

## 1. Introduction

The Expanded Program for Immunization (EPI) in Indonesia is falling short of the World Health Organization (WHO) and the United Nations Children’s Fund (UNICEF) target to reach 90% of children under the age of 1 nationwide and at least 80% in every province by 2020 (1, 2). The Coronavirus Diseases 2019 (COVID-19) pandemic has dramatically impacted routine immunization performance in Indonesia (3, 4). The national immunization program data showed a decline in the coverage of basic immunization programs from 93.6% in 2020 to 84.5% in 2021, indicating that thousands of children will be at risk of increased morbidity and mortality from the outbreaks of vaccine-preventable diseases (VPDs) (5). Coverage of immunization is at risk because restrictions have already led to temporary closure and service suspensions among integrated public healthcare facilities as the backbone of routine maternal, newborn, and child services in Indonesia. On the other hand, subsequent reports showed that the proportion of immunization services at private healthcare facilities has been growing significantly (6, 7). Despite the transition to universal health coverage, private healthcare facilities still dominate Indonesia’s healthcare system, where 64% of Indonesia’s hospitals are private (8). A recent immunization perception survey in Indonesia showed a high demand for safe and timely vaccination services during the COVID-19 outbreak (5). Respondents strongly supported government policy to continue the immunization services with safety precautions, and private healthcare facilities are preferable places for getting immunization services (5). This preference for private healthcare facilities might be due to the proximity of private healthcare facilities to the people, the constant availability of healthcare professionals at private healthcare facilities, and poor services in some public healthcare facilities (9). This preference for healthcare facilities informed the need to enhance the utilization of the private sector in immunization programs while maintaining the high quality of services provided. In addition, from a global perspective, the private sector performs various tasks and activities to support national immunization programs. In low-and middle-income countries (LMICs), it supports the delivery of immunization services and promotes early acceptance of new vaccines before their introduction and widespread use by the public sector (10).

This study aimed to rapidly assess immunization services at private healthcare facilities in Indonesia using Bandung as the reference city. As the capital of West Java Province, Bandung is considered the most densely populated city in Indonesia, with a density of over 14,000 people per square kilometer (11). The latest basic health research conducted by the Ministry of Health (MoH) in 2018 reported that the complete basic immunization coverage for children aged 12–23 months in this region was only 58% (12). Compared with other regions, this coverage was lower, possibly caused by underreporting data from private healthcare facilities. Hence, the objectives of this exercise were to identify gaps, gather perceptions of relevant stakeholders, and prepare for the scale-up of immunization activities at private healthcare facilities.

## 2. Methods

A review of available data, existing policy, legal review, and published literature was applied as the initial step to analyze the situation of immunization services at private healthcare facilities. In addition, primary data were collected by identifying problems and gaps in vaccine service delivery, human resources, and supply chain management, and delivering questionnaires to 9 of 30 (30%) private healthcare facilities that deliver immunization services in Bandung (13). Applying WHO’s guidelines on Service Availability and Readiness Assessment (SARA) to assess service readiness for childhood routine immunization services at private healthcare facilities, several significant variables were taken into account in the questionnaire by focusing on basic amenities and equipment, such as general characteristics (e.g., service days per month, hours of service in a typical day, staff involved in vaccination, and outreach services available), staff and training (e.g., guidelines for EPI and staff trained in EPI), equipment (e.g., cold boxes/vaccine carriers with ice packs, refrigerators, sharp containers, single-use standard disposable or auto disposable syringes, continuous temperature monitoring devices in the refrigerators, energy sources and power supplies for vaccine refrigerators and immunization cards), and vaccine availability (e.g., current stock and stock-outs in the past 3 months) (14).

Furthermore, a qualitative study was considered by developing framework approaches and conducting interviews with different layers. Applying WHO’s framework on monitoring the immunization system (15), in-depth interviews with mid-level managers were conducted in 9 selected private healthcare facilities, representing a type A hospital (*n* = 1; TAH), type B hospital (*n* = 2; TBH1 and TBH2), type C hospital (*n* = 2; TCH1 and TCH2), vaccination house (*n* = 2; VH1 and VH2), and private clinic (*n* = 2; PC1 and PC2). These respondents comprise private-for-profit (*n* = 7) and private-not-for-profit institutions (*n* = 2). Questions focused on five components of the immunization system: service delivery, vaccine supply, quality, and logistics; surveillance and monitoring; advocacy and communication; and program management (15). Each component has different vital points to be explored during the interview process (see Table 1).

**Table 1:** Framework approach for in-depth interviews with mid-level managers.

Components	Key points
Service delivery	Coverage rate Drop-out rate Existence of a national plan for immunization
Vaccine supply, quality, and logistics	Availability and continuity of services Existence of guidelines on vaccine management, transport management, cold chain, and waste disposal and destruction Cold-chain equipment operating and in good repair Completion and display of cold-chain monitoring charts Existence of inventory of immunization equipment that includes date-of-purchase, functional status, maintenance schedule, and evidence that equipment that has been maintained. Availability and sustainable access to other immunization equipment Vaccine forecasting, vaccine utilization, and wastage monitoring Quality of vaccines, including fully functional National Regulatory Authority or other independent assessment of quality performed, manufacturer viable or vaccines procured from prequalified Implementation of a multi-dose vial policy Completion of a standardized immunization injection safety survey Existence and implementation of policy, plan, and budget on injection safety assessment Type of injection equipment in use Method of injection equipment disposal
Surveillance and monitoring	Completeness and timeliness of routine reporting Completion and display of coverage/drop-out monitoring charts Vaccine-preventable disease (VPD) incidence rate Proportion of cases confirmed by laboratory Mortality rate and case fatality rate System for detecting, investigating, and reporting adverse events following immunization (AEFI) Notified and investigated AEFI Case/outbreak investigation initiated within 48 h of notification Percentage of reported VPD cases with information on age and vaccination status Feedback of data to sub-national levels Supervisory checklists complete Development of monitoring indicators Staff monitor status and stock of supplies, equipment, and consumables
Advocacy and communication	Availability of social mobilization, advocacy, or overall communication plan Availability of specific strategy for hard-to-reach population in immunization policy Existence of clinician advocacy and community mobilization Existence of active community health committees Planning meetings conducted with communities Community mobilizers involved in immunization sessions and outreach Engagement of sectors other than the MoH (e.g., Information, Education, Finance, Development, and Planning) Commitment of a broad range of high-level decision makers (demonstrated by active support and public promotion) Budget for activities, staffing, and materials Availability of adequate and appropriate information, education, and communication (ICE) materials
Program management	Government funding of vaccines for routine immunization and program-recurrent costs for supplies and operations Multiple-year commitment to financing Proportion of planed supportive supervision visits conducted Adequacy of personnel to carry out tasks Adequacy of personnel training Reports on implementation of the plans Assessment of services conducted

Following a framework by Tan et al. on the significant achievements related to immunization advocacy to strengthen the immunization outcomes in private sectors in Indonesia (6), in-depth interviews with healthcare professional organizations and public stakeholders were conducted, focusing on efforts to increase coordination across public and private sectors, to improve service delivery, and to strengthen institutional capacity for advocacy and immunization systems support. As an alternative to get some insights from professional organizations and public stakeholders on these three efforts, interviews were conducted with healthcare workers’ organizations (*n* = 2; Indonesian Doctor Association/IDA and Indonesian Pediatrician Association/IPA), hospital associations (*n* = 2; Indonesian Hospital Association/IHA and Indonesian Private Hospital Association/IPHA), central government, which was represented by Indonesian MoH (*n* = 1; Directorate of Immunization Management/DIM), and local government, which was represented by Bandung District of Health/DoH (*n* = 6; Department of Disease Prevention and Control/DDPC, Department of Healthcare Services/DHS, Department of Human Resources/DHR, Department of Public Health/DPH, and two primary healthcare centers/PHC1 & PHC2).

## 3. Results

### 3.1. Situation analysis

The results showed that most private healthcare facilities (56%) provided essential immunization services for children at >6 h per day and < 25 days per month. The number of vaccination staff the MoH had trained varied from 2 to 16 members of staff. Most private healthcare facilities (67%) applied guidelines for immunization services and developed additional internal guidelines. Regarding vaccine availability, the majority of healthcare facilities confirmed that they have available vaccines (e.g., MR, BCG, polio, pentavalent, PCV, IPV, and hepatitis B vaccine) for essential childhood immunization services at that moment. Only a few healthcare facilities confirmed that they did not have the MR (11%), PCV (11%), and IPV vaccine (33%). In the context of experiencing vaccines being out of stock in the last 12 months, all healthcare facilities mentioned that they had these experiences for MR (22%), BCG (44%), polio (33%), pentavalent (33%), PCV (22%), and IPV vaccines (22%). Most healthcare facilities applied self-procurement for PCV (67%), IPV (56%), and hepatitis B vaccines (56%). In particular, most of them (67%) applied a combination of self-procurement and government programs for MR, BCG, polio, and pentavalent vaccines (see Table 2).

**Table 2:** Results of situation analysis.

Variables		Number (*n*)		Percentage (%)	
*Type of private healthcare facilities*					
Hospital		5		56%	
Non-hospital		4		44%	
*Immunization service*					
Per day	Per month	Per day	Per month	Per day	Per month
3–6 h	<25 days	4	5	44%	56%
>6 h	> = 25 days	5	4	56%	44%
*Staffs in vaccination unit*					
Number of staffs	Trained staffs by MoH	Number of staffs	Trained staffs by MoH	Number of staffs	Trained staffs by MoH
<6 staffs	<6 staffs	3	6	33%	67%
6–10 staffs	6–10 staffs	3	3	33%	33%
>10 staffs	>10 staffs	3	0	33%	0%
*Guideline for immunization services*					
Using guideline	Type of guideline	Using guideline	Type of guideline	Using guideline	Type of guideline
No	Internal guideline	3	4	33%	67%
Yes	MoH’s guideline	6	2	67%	33%

Vaccine availability																				
MR	BCG	Polio	Penta	PCV	IPV	HepB	MR	BCG	Polio	Penta	PCV	IPV	HepB	MR	BCG	Polio	Penta	PCV	IPV	HepB
Yes	Yes	Yes	Yes	Yes	Yes	Yes	8	9	9	9	8	6	9	89%	100%	100%	100%	89%	67%	100%
No	No	No	No	No	No	No	1	0	0	0	1	3	0	11%	0%	0%	0%	11%	33%	0%
*Vaccine stock out in the last 12 months*																				
*MR*	*BCG*	*Polio*	*Penta*	*PCV*	*IPV*	*HepB*	*MR*	*BCG*	*Polio*	*Penta*	*PCV*	*IPV*	*HepB*	*MR*	*BCG*	*Polio*	*Penta*	*PCV*	*IPV*	*HepB*
Yes	Yes	Yes	Yes	Yes	Yes	Yes	2	4	3	3	2	2	0	22%	44%	33%	33%	22%	22%	0%
No	No	No	No	No	No	No	7	5	6	6	7	7	9	78%	56%	67%	67%	78%	78%	100%
*Vaccine procurement*																				
MR	BCG	Polio	Penta	PCV	IPV	HepB	MR	BCG	Polio	Penta	PCV	IPV	HepB	MR	BCG	Polio	Penta	PCV	IPV	HepB
SP	SP	SP	SP	SP	SP	SP	2	2	2	2	6	5	5	22%	22%	22%	22%	67%	56%	56%
GP	GP	GP	GP	GP	GP	GP	1	1	1	1	0	1	0	11%	11%	11%	11%	0%	11%	0%
C	C	C	C	C	C	C	6	6	6	6	3	3	4	67%	67%	67%	67%	33%	33%	44%

Equipment		
*Cold box/vaccine carrier with ice packs*		
Yes	9	100%
No	0	0%
*Refrigerator*		
Yes	9	100%
No	0	0%
*Sharp container*		
Yes	7	78%
No	2	22%
*Single use standard/auto disposable syringe*		
Yes	8	89%
No	1	11%
*Continuous temperature monitoring device*		
Yes	9	100%
No	0	0%
*Energy source and power supply*		
Yes	7	78%
No	2	22%
*Immunization card*		
Yes	9	100%
No	0	0%

MoH, Ministry of Health; MR, Measles Rubella; BCG, Bacillus Calmette–Guérin/TB vaccine; PCV, Pneumococcal Conjugate Vaccine; IPV, Inactivated Poliovirus Vaccine; HepB, Hepatitis B; SP, self-procurement; GP, government program; and C, combination of self-procurement and government program.

### 3.2. In-depth interviews with mid-level managers in healthcare facilities

#### 3.2.1. The lack of coordination across public and private sectors

- Underreporting immunization coverage data

“I think 58% is underrated. Many private hospitals might not report their data (TBH2).”

“This number is too low. The major possibility is data from independent medical practice have not been included (VH1).”

- Unclear report on vaccine utilization

“To our knowledge, there is no mandatory to report the use of vaccines to the DoH. (TCH1).”

“Regarding the use of vaccines that are self-procured and obtained from the government, reports have to be submitted routinely every month to the DoH (VH1).”

“We only report the use of vaccines procured by the government (PC2).

- Various types of agreements between the government and private sectors allow private healthcare facilities to use vaccines procured by the government

“We have a written contract with the DoH to get vaccines from the primary healthcare center (PC2).”

“There is an official document, and we are also encouraged to send monthly report [sic] to the primary healthcare center that gives us vaccines (TBH1).”

“We do not have any contract or cooperation documents with the DoH or the primary healthcare center (TCH1).”

- Differences in the frequency of immunization services monitoring

“The DoH, through the primary healthcare center, conducted a routine monitoring of immunization services and vaccine supply chain in our healthcare facility (TBH2).”

“The primary healthcare center supervised and monitored our immunization services and vaccine supply chain management only once at the beginning (VH2).”

“When the DoH visited our healthcare facility for supervision and monitoring, they only asked about immunization technicalities, such as the standard operating procedure of vaccine cold-chain (TCH1).”

- Various types of coverage, drop-out rate, and incidence of VPDs monitoring

“Monitoring coverage and the drop-out rate are done through the patients' vaccine books. We send parents a reminder of the vaccination schedule (PC1).”

“*We use a vaccine diary or passport to maintain coverage and minimize drop-out rate. In particular, most doctors and their nurses have initiatives to ascertain patients' attendance for vaccination one day before the schedule of appointment* (TAH).”

“We monitor the incidence of VPDs through updated news from media, data on the use of vaccines, and patient’s medical record (TBH1).”

“We do routine monitoring related to the incidence of VPDs. We have an interesting story during the COVID-19 pandemic where we found a significant increase of PCV immunization requests from patients (TCH 2).”

#### 3.2.2. The need for immunization service delivery improvement at private healthcare facilities

- Vaccine availability and the number of patients’ visits are critical indicators of immunization services at private healthcare facilities

“All private healthcare facilities confirmed that there are two key indicators of their immunization services, such as vaccine availability and number of patients’ visits.”

“We believe that our brand is strongly associated with good services, and it helps us deliver immunization services as well (TCH1).”

- Impact of the pandemic on routine immunization services

*“The availability of vaccines is limited because of the pandemic, such as pentavalent and polio vaccine (PC1).”*

*“There is a significant decline in the number of hospital visits, possibly due to the stigma of visiting hospital is not safe, so many patients turned to private clinics for getting immunization services* (TBH2).”

- Impact of national immunization plan, such as PCV, which will be included in the national program in 2024, on immunization services at private healthcare facilities

*“It will have an impact on reducing our revenue, but we always commit to supporting the national immunization programs in achieving the targeted coverage (TBH1).”*

*“Depending on the parents' choices between getting the free vaccine from public healthcare facilities or visiting private healthcare facilities with additional costs for certain reasons (TBH2).”*

- Availability and sustainability of public immunization services need to be improved

*“Up to now, we can request routine vaccines from the DoH. If they have vaccines out of stock, we do self-procurement through official distributors (TAH)”.*

*“Monitoring minimal stock is crucial to avoid out of stock (TCH2).”*

*“Patients make appointment [sic] first, and the availability of vaccines will be ascertained before they come (VH2).”*

- Guidelines for vaccine management, transportation management, cold-chain, and waste disposal should be updated regularly

*“We always follow the government guidelines (PC2).”*

*“We use a guideline developed by our central office (VH2).”*

*“We update our guidelines from materials we receive from related seminars (TCH2).”*

*“Evaluation and monitoring are based on daily usage data (TBH2).”*

- Vaccine quality and safety should be monitored routinely

*“Routine monitoring and evaluation from the DoH are important for us to maintain vaccine quality and safety (TCH2).”*

“*We do a daily refrigerator temperature monitoring twice a day (TBH1).”*

*“Refrigerator temperature is monitored by the engineering division three times a day (TAH).”*

- Detection, investigation, and reporting of adverse events following immunization (AEFI) are important

*“All private healthcare facilities confirmed that they do monitoring [sic] AEFI for 48 hours after immunization. All of them also confirmed that they had no experience on [sic] finding AEFI cases until now.”*

*“To monitor AEFI, patients are asked to wait for 30 minutes after immunization and to do self-monitoring for 48 hours after that (TBH2).”*

- The vaccine procurement plan and multi-dose vial policy should be evaluated

*“The pharmacy unit performs a vaccine procurement plan using consumption method and considering safety stock* (TBH1).”

*“The vaccination unit conducts planning, and estimation is made by considering several existing customers (VH1).”*

*“Multi-dose is more wasted than single-dose, even though it can be anticipated (PC2).”*

*“Multi-dose is less efficient because of higher wastage rate. It is better to use single-dose (TAH).”*

#### 3.2.3. The urgency to strengthen institutional capacity for advocacy and immunization systems support

- Social mobilization, advocacy, and communication of immunization services need to be expanded on

*“In addition, we have routine seminars as a media to promote our immunization services and to communicate with the society (TCH2).”*

*“To expand our communication with hard-to-reach population* [sic]*, we are also active in social media and regular webinars or workshops in collaboration with the primary healthcare center and other stakeholders (TBH2).”*

*“Materials for social mobilization, advocacy, and communication of immunization services are arranged by immunization unit* [sic] *and supervised by medical doctors (VH2).”*

- Different types of budgeting for social mobilization, advocacy, and communication of immunization services

*“We do not have specific* [sic] *budget for social mobilization, advocacy, and communication of immunization services planning because it is already included in our routine activities (PC1).”*

*“There is a specific budget for social mobilization, advocacy, and communication of immunization services that is arranged by two divisions of marketing and public relations (TBH1).”*

- Government supports social mobilization, advocacy, and communication of immunization services

*“There is no direct involvement or support from the MoH, DoH, or primary healthcare centers for social mobilization, advocacy, and communication of immunization services in our healthcare facility (VH1).”*

*“The government only supports monitoring and procuring vaccines for the public program through the DoH and primary healthcare centers. Until now, no government supports for* [sic] *social mobilization, advocacy, and communication of immunization services (TCH1).”*

- Government funding of vaccines for routine immunization and program-recurrent costs for supplies and operations

*“There is no special funding from the government because all vaccines in our healthcare facility are self-procured independently (VH2).”*

*“We apply a combination of self-procurement and government programs for childhood vaccination programs. We request some vaccines from the DoH. When our requested vaccines are out of stock in the primary healthcare center, we should have active initiatives to follow up until these vaccines are ready in stock (TBH1).”*

- Adequacy of personnel to carry out tasks and personnel training

*“We sent human resources to join the training organized by the MoH approximately 2-3 years ago* (PC2).”

*“Our healthcare facility has the initiative to conduct regular in-house training for immunization services led by a medical doctor (TAH).”*

- Regular reports on the implementation of the plans and assessment of services conducted

*“There is no direct support from the government to our healthcare facility on vaccine procurement planning. The only support is when they confirm the availability of our requested vaccines (PC1).”*

*“Regarding immunization services' plans in our healthcare facility, including vaccine procurement, no DoH involvement exists. In addition, they never ask for reports on implementing plans (TCH2).”*

*“Since we also request several vaccines to the primary healthcare centers, it is important for them to ascertain vaccine availability and to minimize potential out-of-stock (TAH).”*

### 3.3. In-depth interviews with professional organizations and public stakeholders

#### 3.3.1. Coordination across public and private sectors needs To Be increased

*“It is crucial to have a legal agreement between the government and private sectors, which will facilitate private healthcare facilities to get vaccines from the government. This agreement should cover the mandatory of private healthcare facilities to submit a monthly report on the utilization of vaccines for self-procurement and government programs. AEFI reporting should also be considered, including the flow to report AEFI (IHA).”*

*“Reporting the use of vaccines to the government is mandatory for all healthcare facilities. In the context of government support to private facilities on immunization services, the DOH should provide regular training programs that focus on the distribution, supply chain, and procurement of vaccines. Monitoring of the implementation of vaccinations at private facilities should also be routinely conducted (IPHA).”*

*“For general practitioners or pediatricians who practice independently and carry out vaccination programs, we encourage them to submit regular reports to the DoH regarding the utilization of vaccines. The government should have specific standard operating procedures and reporting formats to create a more practical reporting system than the current situation. Providing a user-friendly online platform also will be an additional benefit (IDA).”*

*“The MoH should have a clear regulation on whether private healthcare facilities should communicate with the DoH or with the nearest primary healthcare center to get vaccines and report the use of vaccines. The possibility that routine vaccinations are not fully reported because of unclear regulations can be minimized. Additionally, there is an urgent need for more comprehensive standard operating procedures for vaccine distribution, implementation, and administration of childhood vaccinations at private healthcare facilities (IPA).”*

*“To increase coordination across public and private sectors, the technical guideline of immunization services at private healthcare facilities will be published soon. This guideline is based on the Minister of Health Regulation number 12 of 2017 concerning immunization. The MoH also will increase the number of vaccination training and intensify monitoring-supervision for private healthcare facilities (DIM).”*

*“In the contract document between the primary healthcare center and private healthcare facility, it has been mentioned that one of the private healthcare facility’s obligations is reporting the utilization of vaccines regularly every month, and one of their rights is receiving vaccines from the primary healthcare center. We supervise and monitor at least once a year for private clinics only. For hospitals, we only do if there are major issues or concerns (PHC1).”*

*“To our knowledge, we only receive reports on the use of vaccines from clinics and midwives. For private hospitals, they send the report to the DoH directly. We supervise and monitor private healthcare facilities, if required only, based on an assignment order from the DoH. The contract document between the primary healthcare center and private healthcare facility is crucial as the legal form of our cooperation, and it should be managed by the DoH (PHC2).”*

*“In the local government regulation, it has been stated that all private healthcare facilities have to report the use of vaccines to the DoH through the primary healthcare center. Both private hospitals and clinics should follow this point in Bandung. For better coordination across public and private sectors, all healthcare facilities must have a contract document with the DoH (DHS).”*

*“Following Mayor’s regulation number 1 of 2020 concerning regional health systems, article 47 mentioned that private healthcare facilities must report immunization activities to the DoH as the consequence of receiving DoH’s support in vaccines (DPH).”*

#### 3.3.2. Service delivery should be improved significantly

*“To improve immunization service delivery, private hospitals can apply several efforts. Firstly, the quality of vaccination data should be improved. Secondly, an automatic system of vaccination schedule reminders is important to maintain coverage and minimize drop-out rates. Last, information about common AEFI cases, such as low-grade fever, should be delivered clearly to the parents (IHA).”*

*“Most private hospitals have realized that vaccination is one of the main good services. They already have initiatives to optimize this potential revenue to provide optimal immunization services through several innovative approaches (IPHA).”*

*“The success story of our social insurance program, BPJS P-Care, can be adopted for vaccination programs. Developing a one-stop-service application is important to improve service delivery for immunization programs in public and private healthcare facilities (IDA).”*

*“Currently, patients have two alternatives to obtain immunization services from public or private healthcare facilities. They can choose their preference based on their needs and economic factors. In this case, the government has the same responsibilities to improve the service delivery of immunization programs in public and private healthcare facilities (IPA).”*

*“Private healthcare facilities are encouraged to apply lean management, which is an approach to create additional values by optimizing resources, such as creating a stable inventory workflow to ascertain vaccine availability and to avoid out-of-stock. Moreover, an information technology system can be considered as the major supporting system (DIM).”*

#### 3.3.3. Institutional capacity for advocacy and immunization systems support is required to be strengthened

*“There should be comprehensive monitoring and supervision from the DoH to healthcare facilities, such as detailed SOPs to maintain the quality, safety, and efficacy/effectiveness of vaccines. Additionally, healthcare facilities should provide regular education programs to the patients and communities about the importance of vaccinations and potential AEFI. The latest recommendation from the Indonesian Doctor Association and the Indonesian Pediatrician Association can be used as the major references (IHA).”*

*“Strengthening institutional capacity for advocacy and immunization systems support can be initiated by enhancing the private hospitals’ awareness to regularly report the use of vaccines. Under-reported data from private hospitals may cause low vaccination coverage. On the other hand, the government should conduct routine supervision and monitoring of these facilities (IPHA).”*

*“Even though private healthcare facilities have followed technical guidelines arranged by the MoH, a comprehensive mapping of their resources and needs is necessary to be conducted by the DoH to strengthen institutional capacity for advocacy and immunization systems support effectively. In particular, the development of online vaccination reporting platform for private healthcare facilities should be accelerated (IDA).”*

*“Series of training for vaccinators at private healthcare facilities are important to improve their competence following the MoH’s regulation. This approach can strengthen institutional capacity for advocacy and immunization systems support. A comprehensive monitoring system is also crucial to avoid the misuse of vaccines (IPA).”*

*“Ideally, supervision and monitoring of private healthcare facilities are routinely conducted through face-to-face visits. Nevertheless, the pandemic has impacted intensifying these activities (DIM).”*

*“Before the pandemic, supervision and monitoring were routinely carried out with limited human resources, specifically for vaccine cold chain. The urgency of contract documents between private healthcare facilities with the DoH should be reviewed because childhood immunization is a national program. Implementation of COVID-19 vaccination can be used as a reference case when contract document was not required (DDPC).”*

*“Given limited human resources in routine immunization programs, it is crucial to develop an application that can assist healthcare facilities in reporting data and the DoH officers to supervise and monitor (DHR).”*

## 4. Discussion

Immunization services at public healthcare facilities in Indonesia were disrupted at 65–90% because of the pandemic (3). In contrast with public healthcare facilities, the demand for immunization services at private healthcare facilities has been increasing significantly in the last 2 years. This situation occurred in many countries, highlighting the need for various contributions from the private sector, including private healthcare facilities. In Indonesia, childhood immunizations are a package of essential health services provided and financed by the government. The government’s ability to deliver these services is directly affected by governance, administrative capacity, and economic factors (10). In particular, health financing, infrastructure, and competing health priorities challenge the desire to provide more comprehensive immunization services (16). Hence, the role of private healthcare facilities in vaccination coverage and practices should be accelerated by enhancing interaction between public and private sectors, the level of monitoring, and the degree of regulations imposed on private healthcare facilities (17).

Our study is the first to assess immunization services at private healthcare facilities in Indonesia. Nevertheless, it has several limitations, and one of the significant limitations is about setting of the study. Firstly, we only considered one respondent from one institution in our in-depth interviews. To ascertain that the critical person is enough to give a complete account of the situation of interest, we listed and ranked potential participants who could meet our purposes. Secondly, we focused our study on Bandung, the capital of West Java Province, the most populous province in Indonesia with a relatively low childhood vaccination coverage (12). Using this such a region as the case study, we expect the results of this study to be one of the references to enhance the role of private healthcare facilities in delivering immunization services. The situation analysis showed that private healthcare facilities had provided sufficient time for essential childhood immunization services with adequate staff. However, the limited staff the MoH has trained remains a programmatic problem. Furthermore, private healthcare facilities have used the MoH’s guidelines and additional internal guidelines for immunization services as the primary references, such as providing complete and reliable equipment. Vaccine availability at private healthcare facilities is manageable, with the out-of-stock vaccine level remaining acceptable.

The qualitative evaluation provided a critical view of immunization services at private healthcare facilities by gathering perceptions of healthcare workers and other relevant stakeholders. Applying WHO’s framework for monitoring the immunization system (14), we collected information from mid-level managers at private healthcare facilities by focusing on service delivery, vaccine supply, quality, and logistics; surveillance and monitoring; advocacy and communication; and program management. This evaluation highlighted three key findings: the lack of coordination across public and private sectors, the need for immunization service delivery improvement at private healthcare facilities, and the urgency to strengthen institutional capacity for advocacy and immunization systems support. In the context of coordination across public and private sectors, we found several interesting findings, such as the importance of legal agreements between the DoH and private healthcare facilities and the urgency for private healthcare facilities to report the use of vaccines from self-procurement and government programs. Another critical issue is immunization service delivery at private healthcare facilities. All private healthcare facilities confirmed that there are two critical indicators of their immunization services, such as vaccine availability and the number of patient visits. As most private healthcare facilities apply a combination of vaccine self-procurement and government programs, support from the government in terms of vaccine availability is significant. When private healthcare facilities can avoid out-of-stock vaccines, the performance of immunization services can be maintained, and the number of patient visits can be increased simultaneously. The last concern is about institutional capacity for advocacy and immunization systems support. Private healthcare facilities require regular DoH supervision and monitoring to improve immunization services, including vaccine supply chain management continuously.

By conducting in-depth interviews, we gathered insights from healthcare workers’ organizations, hospital associations, and both central and local government. Feedback from professional organizations and public stakeholders is required to find out solutions related to those findings. Several promising alternatives could be identified. Firstly, the government should publish a comprehensive technical guideline for immunization services at private healthcare facilities immediately to increase coordination across public and private sectors (18, 19). Even though several central and local government regulations have been launched, they should have considered technical and practical issues. Secondly, technology interventions to develop one-stop-service applications can be used as an alternative to improve service delivery for immunization programs in public and private healthcare facilities (20, 21). Lastly, comprehensive monitoring and supervision must be conducted regularly through more detailed SOPs to maintain the quality, safety, and efficacy/effectiveness of vaccines. Given limited human resources, the Internet of Things can assist healthcare facilities in reporting data and the DoH officers in supervision and monitoring (22, 23).

All countries worldwide have variable degrees of government engagement with the private sector to deliver immunization services. In most LMICs, publicly funded immunization services are mainly provided by public healthcare facilities, but the more significant contribution from private healthcare facilities to deliver these services is essential (24, 25). It has been known that private sector engagement can add value to the health system at various levels, including increased access to skills and expertise, operational efficiencies, increased innovation, shared risk, and allowing the government to focus on its core competencies (24, 26). This engagement is significant for Indonesia as a country with limited resources to achieve national health and vaccination goals (24). More effective engagement between the public and private healthcare sectors could improve the performance of health systems by providing better policies, regulations, information sharing, and financing mechanisms (27). If private healthcare facilities already provide a significant proportion of childhood vaccinations, engagement should be focused on service quality issues. If they do not contribute a significant proportion of vaccinations, a potential role for them to expand the reach of public healthcare facilities should be accelerated. Hopefully, this study could assist the stakeholders in the decision-making process related to improving immunization services in Indonesia.

## Data availability statement

The raw data supporting the conclusions of this article will be made available by the authors, without undue reservation.

## Author contributions

AS and AD: conceptualization. AS and RA: methodology. AS and NZ: software, formal analysis, investigation, writing—original draft preparation, and project administration. NZ and RA: validation. RA and AD: resources, writing—review and editing, and supervision. All authors contributed to the article and approved the submitted version.

## Funding

This study was funded by Clinton Health Access Initiative (CHAI) Indonesia.

## Conflict of interest

The authors declare that the research was conducted in the absence of any commercial or financial relationships that could be construed as a potential conflict of interest.

## Publisher’s note

All claims expressed in this article are solely those of the authors and do not necessarily represent those of their affiliated organizations, or those of the publisher, the editors and the reviewers. Any product that may be evaluated in this article, or claim that may be made by its manufacturer, is not guaranteed or endorsed by the publisher.
